# Early interferon lambda production is induced by double-stranded RNA in iPS-derived hepatocyte-like cells

**DOI:** 10.1093/oxfimm/iqae004

**Published:** 2024-06-06

**Authors:** Vasile Mihai Sularea, Ruchi Sharma, David C Hay, Cliona O’Farrelly

**Affiliations:** School of Biochemistry and Immunology, Trinity Biomedical Sciences Institute, Trinity College Dublin, 152 - 160 Pearse St, Dublin, D02R590, Ireland; Stemnovate LTD, Cambridge, Maia Building 270, Babraham Research Campus, Cambridge, CB223AT, United Kingdom; Institute for Regeneration and Repair, Centre for Regenerative Medicine, University of Edinburgh, 5 Little France Drive, Edinburgh, EH16 4UU, United Kingdom; School of Biochemistry and Immunology, Trinity Biomedical Sciences Institute, Trinity College Dublin, 152 - 160 Pearse St, Dublin, D02R590, Ireland; School of Medicine, Trinity College Dublin, 152 - 160 Pearse St, Dublin, D02R590, Ireland

**Keywords:** Hepatocyte, liver, RNA sensing, interferon, antiviral response

## Abstract

Hepatotropic viruses are amongst the most ubiquitous pathogens worldwide, causing significant morbidity and mortality. As hepatocytes are among the primary targets of these viruses, their ability to mount early effective innate defence responses is of major research interest. Interferon lambda (IFNL) is produced early in response to viral stimulation in other cell types, but hepatocyte production of this interferon is little investigated. Due to the difficulty and significant costs in obtaining and culturing human primary hepatocytes, surrogate systems are widely sought. Here we used induced pluripotent stem (iPS)-derived hepatocyte-like cells (HLCs) to investigate hepatic IFNL expression in response to viral-like ligands. We demonstrate that hepatocytes rely on cytoplasmic pattern recognition receptors (PRRs) such as Protein Kinase RNA-dependent (PKR) and retinoic acid-inducible gene-I (RIG-I)-like receptors (RLR) for the detection of double stranded RNA**.** Stimulation of HLCs by viral-like RNA ligands activating cytosolic RNA sensors resulted in thousand fold increase of type III interferon gene expression. These results are in contrast with type I IFN expression, which was induced to a lower extent. Concomitant induction of interferon stimulated genes, such as interferon-stimulated gene 15 (ISG15) and CXCL10, indicated the ability of HLCs to activate interferon-dependent activity. These results demonstrate that HLCs mount an innate antiviral response upon stimulation with viral-like RNA characterized by the induction of type III IFN.

## Introduction

The liver is among the primary targets of some of the most pathogenic viruses in humans, including Hepatitis A-E, Yellow fever, and Dengue virus. Like other cells, hepatocytes detect viral RNA via a range of pattern recognition receptors (PRRs) including RIG-I-like receptors (RLRs), Toll-like receptor 3 (TLR3), and Protein Kinase RNA-dependent (PKR), all of which have been shown to be critical in hepatic antiviral response [1, 2]. While TLR3 is located in the endosomal compartment, RLRs and PKR are cytoplasmic PRRs. Ligation of these receptors induces production of type I and III interferons (IFNs), as well as expression of pro-inflammatory cytokines [3]. By binding to their cognate receptors, IFNs activate an intracellular signalling cascade that ultimately upregulates interferon stimulated gene (ISGs), the effectors of the antiviral response [4].

Type I and III interferons are critical immune messenger molecules produced upon viral infection. IFNs are part of the class II cytokine family, which comprehends interleukin 10 (IL-10)-related cytokines. Type I IFNs include different subtypes in human and mice, 13 and 14 respectively, IFN-α subtypes, a single IFN-β, IFN-ε, IFN-κ, IFN-ω (in humans), and IFN-ζ (in mice). Type III IFNs (also known as the IFN-λ family) consists of 4 genes in humans, IFNL1 (encoding for IL-29 protein), IFNL2 (IL28-A), IFNL3 (IL-28B), and IFNL4 which is a pseudogene in many population and has been associated with HCV clearance [5]. While all type I IFNs signal through a shared heterodimeric receptor (IFNAR), formed by IFNAR1 and IFNAR2 subunits, type III IFN receptor is comprised of IFNLR1 (also known IL28Rα) and IL10Rβ. By binding to their cognate receptors, type I and III interferons signal through the JAK/STAT pathway to induce an antiviral state by activating expression of ISGs, involved in the antiviral responses [6]. Whereas almost all nucleated cells respond to type I interferon by expressing the IFNAR receptor, type III interferons are produced by and target mainly mucosal epithelial cells and hepatocytes [3]. Interestingly, while type III IFN receptors are expressed by human hepatocytes, they are not expressed by murine hepatocytes [7, 8]. The species-specific expression raises severe limitations to studies of the hepatic innate antiviral response in mice. Thus, the development of a human hepatic model that mimics primary human hepatocytes is needed in order to explore the human hepatic innate antiviral activity.

Human hepatoma cell lines have been widely used to study liver function, metabolism and antiviral activity *in vitro*. Among these, HepG2 and Huh7 are the two most commonly used cell lines and have been extensively used as models for viral infection [9]. In particular, Huh7 cells, and its subclonal line Huh7.5 (which carries a mutation on DDX58, the RIG-I encoding gene), have been used by several groups as models for HCV infection [10–13]. However, being tumour-derived, these cell lines have significant limitations in mimicking physiological hepatic metabolism and activity [9, 14]. Other human hepatic cellular models are therefore sought. Among these, human induced pluripotent stem (iPS)-derived hepatocyte-like cells (HLCs) have been shown to be useful and reproducible models for hepatic drug metabolism, hepatic steatosis, and viral infection [15–19]. Since HLCs are derived from primary cells, they are not transformed, as in the case of hepatic cell lines. Thus, mimicking the unique metabolic features of hepatocytes, HLCs are a valid surrogate model for studying human primary hepatocyte behaviour during viral infection [19].

In the present study, using HLCs we sought to determine whether human hepatocytes respond to viral pathogen-associated molecular patterns (PAMPs) through an IFN-based antiviral response. We found that cytoplasmic PRR agonists drive type I and III interferon production with massive over-production of type III interferon compared with type I. HLCs also produced pro-inflammatory cytokines and interferon stimulated gene expression in response to viral ligand stimulations. Production of type III interferon with an earlier kinetic and a stronger induction when compared to type I IFN may be critical to protecting the liver early in viral infection.

## Materials and methods

### iPS-derived hepatocyte-like cell differentiation

**Figure 1.**
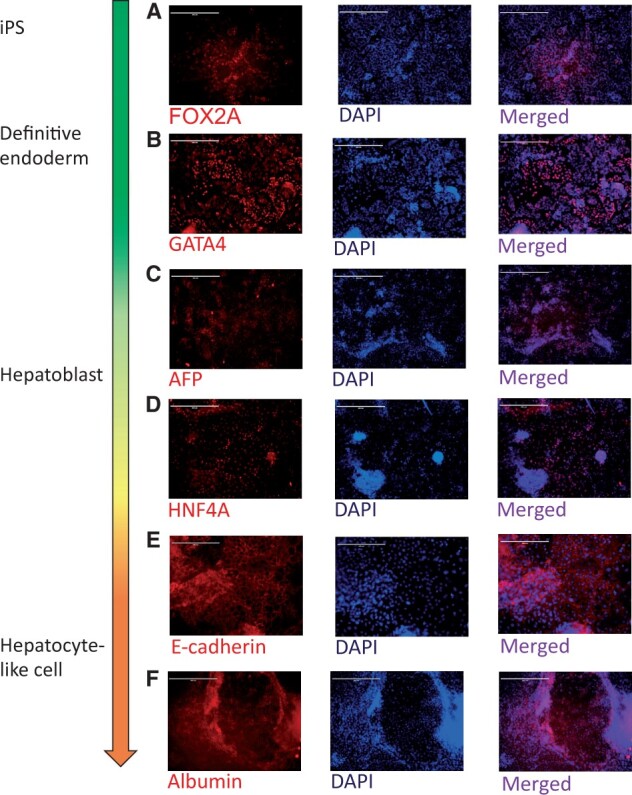
Differentiation of iPSCs into hepatocyte-like cells. iPSCs (induced pluripotent stem cells) were differentiated into hepatocyte-like cells (HCLs) through three different stages. Cells were stained for every differentiation stage. (**A and B**) At the definitive endoderm, cells were stained for FOX2A and GATA4. (**C and D**) At the Hepatoblast stage, cells were stained for alpha-fetoprotein (AFP) and hepatic nuclear factor 4-α (HNF4−α). (**E and F**) At the last stage, the hepatocytes-like cells (HLCs) were stained for E-cadherin and albumin. Cells were counterstained with DAPI for nuclear DNA.

Induced pluripotent stem cells were differentiated into HLCs according to an established protocol [20]. The human embryonic stem cell line H9 (WA09, WiCell) and induced pluripotent stem cells obtained from skin biopsies were seeded in 24-well plate formats (10^5^ cells/well) and differentiated into HLCs through 4 different stages, pluripotent stem cells, definitive endoderm, hepatic progenitors, and hepatocyte-like cells (HLCs) (Stemnovate). Stem cells were cultured on Lamina 521 (5 µg/ml) (Biolamina) in mTESR until reaching 80% confluency. One day prior to differentiation, cells were seeded and treated overnight with 10 µM Rock Inhibitor. The endoderm stage was induced by RPMI 1x supplements with 2% B27, 100 ng/ml Activin A, and 50 ng/ml Wnt3A for 3 days. Subsequently, for the Hepatoblast differentiation, cells were cultured in KO DMEM, serum replacement, 0.5% Glutamax, 1% non-essential amino acids, 0.2% β-mercaptoethanol, and 1% DMSO for 5 days and it was renewed every second day. For the differentiation in hepatocyte-like cells, cells were cultured for further 10 days in HepatoZYME containing 1% Glutamax, 20 ng/ml Hepatocyte Growth Factor, 10 ng/ml oncostatin, and 10 µM hydrocortisone. Media was renewed every second day. Cells were collected on the last day of each stage and stained for specific markers in order to confirm the differentiation process. Expression of the transcriptional factors FOX2A (Fig. 1B) and GATA4 were used as markers for the definitive endoderm stage. Hepatic progenitors were positive for alpha-fetoprotein (AFP) and hepatic nuclear factor 4-alpha (HNF4A) Albumin (Fig. 1F) and E-cadherin were used to confirm the mature hepatocyte-like cell stage.

### Stimulation of HLCs with viral mimics

HLCs were stimulated with 25 µg/ml of poly I: C, 5 µg/ml of transfected poly I: C and 1 µg/ml of 5’-triphosphate hairpin RNA. High molecular weight poly I: C and 5’ triphosphate hairpin RNA (3p-hRNA) were transfected into HLCs using Lipofectamine 2000. The ratio Lipofectamine 2000: transfected RNA was 1 : 2; 1 ml of Lipofectamine was used for every 2 ml of transfected RNA. The mix was prepared starting with two tubes, the first containing Lipofectamine 2000 in serum-free media (OptiMem) and the second containing the RNA in of serum-free media. Both tubes were left for five minutes at room temperature. The contents of the second tube were then transferred into the first tube, mixed and left for at least 20 min to allow formation of the RNA-Lipofectamine complexes. The RNA-lipofectamine was subsequently added to the cells with fresh media.

### RNA extraction and RT-qPCR

Total RNA was extracted using a phenol-chloroform extraction based protocol with TriZol. 1000 ng of RNA per samples was reverse transcribed into cDNA using High-Capacity cDNA Reverse Transcription Kit by Termo Fisher Scientific according to manufacturer’s instruction. PowerUp SYBR Green Master Mix and StepOnePlus Real-time PCR System with StepOne Software v2.3 was used for experiments. A 10 μl reaction volume contained 8 μl pre-made qPCR master mix, primers, nuclease-free water, and 2 μl of cDNA. A non-template control was included in each gene assay to detect molecular contamination. All reactions were carried out in triplicate. The thermocycling program was: 20 s at 95°C, 40 cycles of 95°C for 30 s and annealing temperature of 60° for 30 s, then 95°C for 15 s, 60°C for 1 min and finally 95°C for 15 s. Relative expression (2^−ΔΔCT^) was calculated from the cycle threshold (CT) values for each sample and gene of interest. Ribosomal Protein Lateral Stalk Subunit P0 (RPLPO) a gene demonstrated to be stably expressed during hepatocyte differentiation was used as housekeeper gene in order to calculate changes in relative expression of genes under investigation.

### ELISA

All ELISAs were carried out according to the manufacturer’s instructions. ELISA plates were coated overnight at room temperature with capture antibody (accordingly diluted in PBS, 50 ml/well). The next day, after blocking for 1 h at room temperature (1% bovine serum albumin, BSA in PBS, 50 ml/well), diluted cell supernatants (diluted 1 : 5 in PBS 1% BSA, 50 ml/well) and specific cytokine standard curve were added to each plate in technical duplicate for an overnight at +4°C. The next day, detection antibody (accordingly diluted in PBS 1% BSA, 50 ml/well) was added for 2 h at room temperature. Streptavidin-HRP solution (diluted according to the manufacturer’s instructions in PBS 1% BSA, 50 ml/well) was added for 20 min, before the last step, consisting in addition of the substrate TMB and stopping solution (1 M H_2_SO_4_, 25 ml/well). Between each passage ELISA plates were washed 4 times in washing buffer (PBS + 0.1% Tween-20). Absorbance at 450 nm was quantified using a plate reader. Corrected absorbance values were calculated by subtracting the background absorbance (540 nm). Cytokine concentrations were subsequently obtained by extrapolation from a standard curve plotted in Excel.

### Reagents

 

**Table:** 

Reagent or resources	Source	Identifier
*Antibodies*		
FOX2A	Abcam	Cat# Ab5074
GATA4	Thermo Fisher	Cat# PA1-102
AFP	Thermo Fisher	Cat# K9218
HNF4A	Abcam	Cat# Ab1416
E-cadherin	Abcam	Cat# Ab207327
Albumin	Abcam	Cat# Ab10241
*Chemicals and recombinant proteins*		
High molecular weight poly (I: C)	Invivogen	Cat# tlrl-pic
5′ triphosphate hairpin RNA	Invivogen	Cat# tlrl-hprna
Lipofectamine 2000	Thermo Fisher	Cat# 11668027
Opti-MEM	Thermo Fisher	Cat# 31985062
mTESR	STEMCELL	Cat# 85857
Biolaminin 521	BioLamina	Cat# LN521
Rock inhibitor	Tocris	Cat# 1254
Activin A	Peprotech	Cat# 120-14P
B27	Thermo Fisher	Cat# 17504044
Wnt3A	R&D Systems	Cat# 5036-WN
Hepatozyme	Thermo Fisher	Cat# 1770521
Human hepatocyte growth factor	Peprotech	Cat# 100-39H
Human oncostatin	Peprotech	Cat# 300-10
Hydrocorstisone	Merck	Cat# H0888
*Commercial assays*		
Human IFN-β Duoset ELISA	R&D Systems	Cat# DY814-05
Human IL-29 (IFNL1) Duoset ELISA	R&D Systems	Cat# DY7246
*Primers*		
Ifnb1 forward: 5′- AAACTCATGAGCAGTCTGCA -3′ Ifnb1 reverse: 5′- AGGAGATCTTCAGTTTCGGAGG -3′		
Ifnl1 forward: 5′- TCCAAGCCCACCACAACTG -3′ Ifnl1 reverse: 5′- TGAGTGACTCTTCCAAGGCGT -3′		
Cxcl10 forward: 5′- CCTGCAAGCCAATTTTGTCCA -3′ Cxcl10 reverse: 5′- TGTGGTCCATCCTTGGAAGC -3′		
Isg15 forward: 5′- TTTGCCAGTACAGGAGCTTGTG -3′ Isg15 reverse: 5′- GGGTGATCTGCGCCTTCA -3′		
Mx1 forward:		
5′- GGTGGTGGTCCCCAGTAATG -3′		
Mx1 reverse: 5′- ACCACGTCCACAACCTTGTCT -3′		
Tnf-alpha forward: 5′- TGGCCCAGGCAGTCAGATCA -3′ Tnf-alpha reverse: 5′- GTAGGAGACGGCGATGCGGC -3′		
Rplpo forward: 5′- ACTTGCTGAAAAGGTCAAGGC -3′ Rplpo reverse: 5′- CCAAATCCCATATCCTCGTCCG -3′		

### Quantification and statistical analysis

All data were analysed with GraphPad Prism 10. Data are presented as mean ± SEM Details of specific testes used (unpaired Student’s *t*-test, One-Way and Two-Way ANOVA) are described in Figs. Differences were considered significant when *P* < 0.05.

## Results

### Induced pluripotent stem cell (iPSC) differentiation into hepatocyte-like cells (HLCs)

Induced pluripotent stem cells were differentiated through 3 different stages, definitive endoderm, hepatic progenitors (also known as hepatoblasts), and hepatocyte-like cells (HLCs) (Fig. 1) [20]. The differentiation process took on average 16 ± 1 days. Cell in the definitive endoderm stage were stained for FOX2A and GATA4. Cells in the hepatic progenitor stage were stained for alpha-fetoprotein (AFP) and hepatic nuclear factor 4-alpha (HNF4A). For the last stage, mature hepatocyte-like cells were stained for albumin and E-cadherin. At every stage, cells were counterstained with DAPI for nuclear DNA.

### Hepatocyte-like cells produce significant levels of type III interferon, relying on cytoplasmic PRRs for the detection of dsRNA

**Figure 2.**
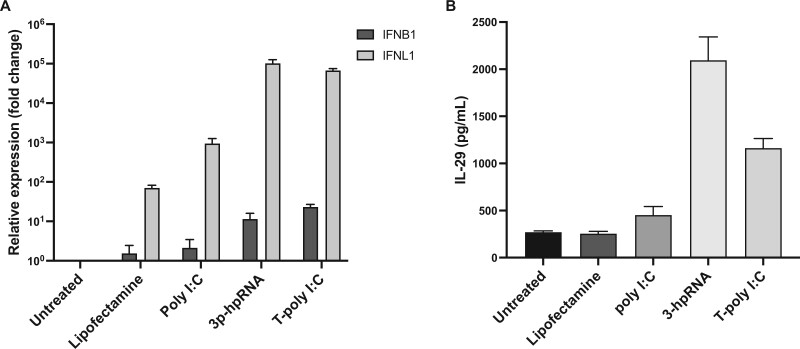
Stimulation with transfected poly I: C and 5’ short-hairpin RNA induce type I and III interferon expression in hepatocyte-like cells (HLCs). HLCs were stimulated for 24 h with non-transfected poly I: C (25 µg/ml), transfected 5’-triphosphate hairpin RNA (3p-hpRNA) (1 µg/ml), and transfected poly I: C (T-poly I: C) (5 µg/ml). Lipofectamine was used as vehicle control for the transfection. (**A**) IFNB1 and IFNL1 relative gene expression were determined by RT-qPCR. (**B**) IL-29 (encoded by the IFNL1 gene) protein levels were measured by ELISA. *N* = 2, mean ± SEM.

We sought to determine whether HLCs were able to mount an innate antiviral response upon stimulations with RNA-based viral-like ligands. HLCs were stimulated with TLR3 (non-transfected poly I: C), RIG-I (5’ triphosphate hairpin RNA), MDA5 and PKR (transfected poly I: C) agonists for 24 h. Upon stimulation with poly I: C, the ligand for TLR3, minimal interferon, either type I or III, was observed. However, stimulation with transfected 5’ triphosphate hairpin RNA (3-hpRNA) and transfected poly I: C (T-poly I: C), activating RIG-I, MDA5 and PKR respectively, induced a significant production of the interferons IFNB1 and IFNL1 (Fig. 2A) and the respective protein IL-29 (Fig. 2B), encoded by the IFNL1 gene.

### Stimulation with cytoplasmic PRRs agonists induces expression of ISGs and pro-inflammatory cytokines in HLCs

**Figure 3.**
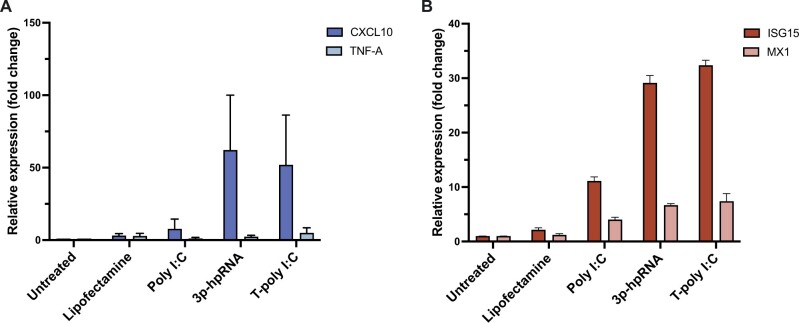
Transfected polyI: C induces expression of ISGs and pro-inflammatory cytokines. HLCs were stimulated for 24 h with non-transfected poly I: C (25 µg/ml), 5’-triphosphate hairpin RNA (3p-hpRNA) (1 µg/ml), and transfected poly I: C (T-poly I: C) (5 µg/ml). Lipofectamine was used as vehicle control for the transfection. (**A**) CXCL10 and TNF relative gene expression were determined by RT-qPCR. (**B**) Interferon stimulated genes ISG15 and MX1 relative gene expression were determined by RT-qPCR. N = 2, mean ± SEM.

We determined whether pro-inflammatory cytokines and interferon stimulated genes were induced in HLCs by stimulation with dsRNA. We analysed expression of the pro-inflammatory cytokines CXCL10 and TNF and observed an increase in CXCL10, while TNF was not induced (Fig. 3A). We further quantified by RT-qPCR the relative gene expression of the interferon stimulated genes ISG15 and MX1. We observed that ISG15, rather than MX1, was upregulated upon stimulation with poly I: C, transfected 5′triphosphate hairpin RNA, and transfected poly I: C (Fig. 3B). In both the cases, transfected 5’triphosphate hairpin RNA and transfected poly I: C, upregulated CXCL10 and ISG15 to a greater extent, when compared with non-transfected poly I: C.

### Time-course study analysis reveals early induction in IFNL1 expression upon dsRNA stimulation in HLCs

**Figure 4.**
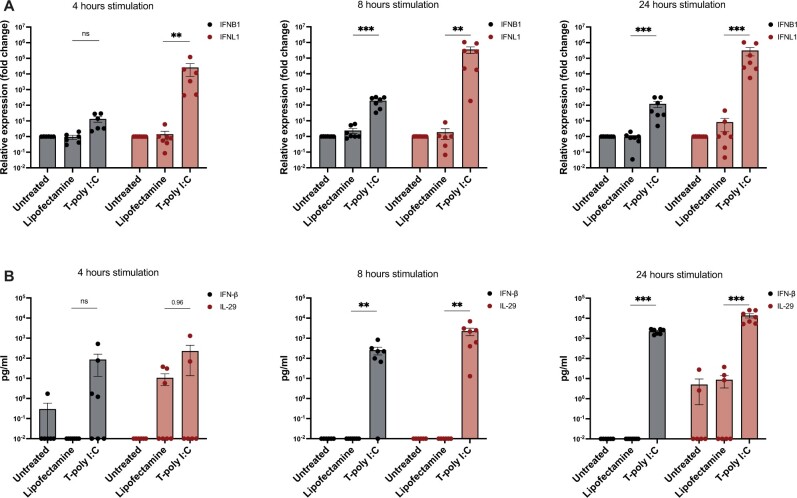
IFN-β and IFNL1 are upregulated upon transfected poly I: C stimulation in HLCs. HLCs were stimulated for 4, 8, and 24 h with transfected poly I: C (T-poly I: C) (5 µg/ml); lipofectamine was used as vehicle control for the transfection. (**A**) IFNB1 and IFNL1 relative gene expression were determined by RT-qPCR. (**B**) IFN-β and IL-29 (encoded by the IFNL1 gene) protein levels were determined by ELISA. N = 7, mean ± SEM, *P* values were calculated using unpaired one-way ANOVA, ns: not significant, *P < 0.05, **P < 0.01, and ***P < 0.001.

We performed a time course-study to determine the optimal time of induction of type I and III IFNs, pro-inflammatory cytokine CXCL10, and the interferon stimulated gene ISG15 upon transfected poly I: C stimulation. We observed an early induction of the antiviral response at 4 h, with an increase in IFNB1 and IFNL1 (Fig. 4A). At the later time points, 8 and 24 h after stimulation, we observed a further increase in the expression of the IFNs with a peak at 24 h (Fig. 4A). These results reflected the protein levels of IFN-β and IL-29, which were significantly upregulated already at 8 h and reached the peak at 24 h after stimulation with transfected poly I: C (Fig. 4B). IFNL1, compared to IFNB1, showed an earlier and stronger induction, with more than a 10 fold change difference in the induction of gene expression as well as protein levels.

**Figure 5.**
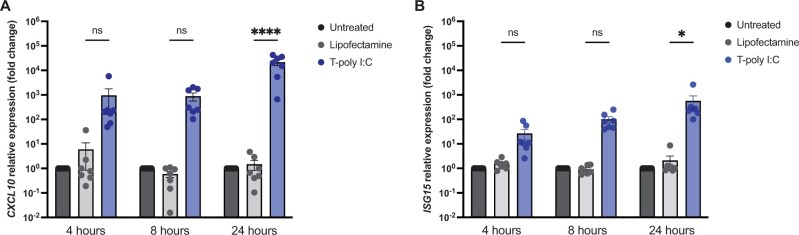
ISG15 and CXCL10 expression are upregulated by transfected poly I: C stimulation in hepatocyte-like cells. HLCs were stimulated for 4, 8, and 24 h with transfected poly I: C (T-poly I: C) (5 µg/ml); lipofectamine was used as vehicle control for the transfection. (**A**) CXCL10 relative gene expression was determined by RT-qPCR. (**B**) ISG15 relative gene expression was determined by RT-qPCR. N = 7, mean ± SEM, p values were calculated using unpaired two-way ANOVA, *P < 0.05, **P < 0.01, and ***P < 0.001.

We further analysed the gene expression of ISGs and pro-inflammatory cytokines. A rapid increase in CXCL10 and ISG15 expression was observed at 4 h, while expression peaked at 24 h (Fig. 5), similar to IFNs expression kinetics.

## Discussion

The majority of hepatotropic viruses are RNA viruses, with few exceptions such as HBV, which replicates through a RNA intermediate therefore activates cellular RNA sensing [21, 22]. Therefore we focused on the innate antiviral response of HLCs induced by dsRNA. We examined the ability of these cells to respond to stimulation with the viral analogue poly I: C by measuring expression of IFNs and genes involved in the innate antiviral response, such as pro-inflammatory cytokines and ISGs. We particularly focused on IFNL1 as it is known to drive antiviral activity in other cells within hours of stimulation but is not as pro-inflammatory as type I interferon [23]. Although we observed an increase in IFNs and ISGs expression upon extracellular dsRNA stimulation, we found that cytosolic delivery of dsRNA caused a greater induction of the antiviral genes indicating that cytoplasmic PRRs, such as PKR and RLRs, are critical to the innate antiviral response of HLCs. Therefore, in our study we focused on the induction of IFN expression upon activation of cytosolic PRRs by transfected dsRNA. Future studies using non transfected dsRNA might help to define the hepatic TLR3-dependent antiviral response.

Our time-course study revealed an early and robust induction of IFNL1, which was higher than IFNB1 expression, both at mRNA and protein levels. While type I IFNs induce antiviral activity in the majority of nucleated cells due to the ubiquitous expression of IFNAR, type III IFNs responsiveness is limited to epithelial barriers such as gastrointestinal, female reproductive, and respiratory tracts as well as liver, placenta, and blood-brain barrier [3]. It is held that type III interferons have an anti-viral role and according to literature mostly based on *in vitro* and cell lines experiments, type III IFNs induce a less potent but similar set of genes to type I IFNs [24, 25]. However, recent studies, based on primary cells and *in vivo* experiments, showed different genes induced by the two IFN families [26]. The kinetics of antiviral genes induced by type I and type III IFNs are distinct; type I IFN-induced ISGs present an early peak and decline, while type III IFNs induce more sustained expression of antiviral genes [27]. Whether this difference is caused by the distinct activity of negative regulatory ISGs like suppressors of cytokine signalling (SOCS) or it is due to other mechanisms is still object of study [28, 29].

In vivo mouse models can be used to study type I and III IFNs differences in respiratory and gastrointestinal tract infections [30, 31]. This is not possible for the liver, since mouse hepatocytes, in contrast to human hepatocytes, do not express the type III IFN receptor IFNLR1 [7, 8]. Primary human hepatocytes are challenging to source and culture successfully therefore surrogate models are needed to dissect differences between the two IFN families. Hepatocyte-like cells have been shown to recapitulate primary hepatocyte characteristics [32]. HLCs bring several improvements compared to human hepatic cells lines, they are derived from primary human cells, thus they are not malignant cells and their genetic background is preserved. Furthermore, HLC metabolism is more similar to primary human hepatocytes compared to hepatic cell lines, which present a highly glycolytic phenotype. We have recently demonstrated that HLCs are capable of mounting an antiviral response upon extracellular and cytosolic dsRNA stimulation [2]. Here, we extend these studies to show that HLCs are potent producers of type III interferon at an early stage of antiviral activity. The ability of HLCs to induce type III IFNs, as shown in this study, highlights the potential role for this cellular model in studying the early innate antiviral response in human hepatocytes. Future studies using HLCs as cellular model for human hepatocytes might help us to identify the main PRRs activated in this cell type upon RNA-sensing during hepatotropic viral infections. Therefore, this would help the development of novel antiviral therapies targeting specific viral sensing pathways.

A recent study showed the therapeutic capacity of pegylated IL-29 for COVID-19 [33]. It has been suggested that IL-29 treatment could be a valid therapeutic option against hepatotropic pathogens, such as HBV and HCV [34, 35]. Moreover, type III interferons specifically activating only the innate antiviral response of hepatocytes, might reduce the adverse effects observed in IFN-α-based treatments [36]. Future studies using HLCs would improve our understanding of the role of type III interferon in the hepatic antiviral response and potentially aid in developing type III interferon-based pharmacological strategies against hepatotropic viruses.

## Data Availability

The data underlying this article will be shared on reasonable request to the corresponding author.
